# Protectin DX promotes epithelial injury repair and inhibits fibroproliferation partly via ALX/PI3K signalling pathway

**DOI:** 10.1111/jcmm.16011

**Published:** 2020-10-24

**Authors:** Jing‐Xiang Yang, Ming Li, Xin Hu, Jia‐Chao Lu, Qian Wang, Shi‐Yue Lu, Fang Gao, Sheng‐Wei Jin, Sheng‐Xing Zheng

**Affiliations:** ^1^ Department of Anesthesia and Critical Care The Second Affiliated Hospital and Yuying Children's Hospital of Wenzhou Medical University Zhejiang China; ^2^ Birmingham Acute Care Research Group Institute of Inflammation and Aging University of Birmingham Birmingham UK

**Keywords:** acute respiratory distress syndrome, apoptosis, fibroblast proliferation, transdifferentiation, type II alveolar cells, wound healing

## Abstract

Acute respiratory distress syndrome/acute lung injury (ARDS/ALI) is histologically characterized by extensive alveolar barrier disruption and excessive fibroproliferation responses. Protectin DX (PDX) displays anti‐inflammatory and potent inflammation pro‐resolving actions. We sought to investigate whether PDX attenuates LPS (lipopolysaccharide)‐induced lung injury via modulating epithelial cell injury repair, apoptosis and fibroblasts activation. In vivo, PDX was administered intraperitoneally (IP) with 200 ng/per mouse after intratracheal injection of LPS, which remarkedly stimulated proliferation of type II alveolar epithelial cells (AT II cells), reduced the apoptosis of AT II cells, which attenuated lung injury induced by LPS. Moreover, primary type II alveolar cells were isolated and cultured to assess the effects of PDX on wound repair, apoptosis, proliferation and transdifferentiation in vitro. We also investigated the effects of PDX on primary rat lung fibroblast proliferation and myofibroblast differentiation. Our result suggests PDX promotes primary AT II cells wound closure by inducing the proliferation of AT II cells and reducing the apoptosis of AT II cells induced by LPS, and promotes AT II cells transdifferentiation. Furthermore, PDX inhibits transforming growth factor‐β_1_ (TGF‐β_1_) induced fibroproliferation, fibroblast collagen production and myofibroblast transformation. Furthermore, the effects of PDX on epithelial wound healing and proliferation, fibroblast proliferation and activation partly via the ALX/ PI3K signalling pathway. These data present identify a new mechanism of PDX which targets the airway epithelial cell and fibroproliferation are potential for treatment of ARDS/ALI.

## 1 INTRODUCTION

Acute respiratory distress syndrome/acute lung injury (ARDS/ALI) is histologically distinguished by extensive destruction of the alveolar barrier due to significant alveolar epithelial cell injury and apoptosis, excessive fibroproliferation responses, and the accumulation of neutrophils in the lung.^1^, ^2^, ^3^ The alveolar epithelial cells are primary targets during ARDS.^4^ The degree of epithelial injury constitutes a critical prognostic marker of ARDS.^3^ Thus, timely repair of the alveolar epithelial barrier plays a key role in the resolution of ARDS/ALI.^5^

Upon injury of the alveolar epithelium leads to denudation of the epithelial basal lamina.^6^ AT2 cells, as the progenitor cells for the alveolar epithelium, proliferate, spread on the denuded basal lamina and transdifferentiate into ATI cells. AT2 cells injury with dysregulated and delayed repair activates interstitial fibroblasts and starts to produce excessive amounts of ECM components resulting in fibrosis finally.^7^ Significant fibroproliferative responses are associated with bad prognosis such as prolonged mechanical ventilation and high mortality.^8^ The studies also suggested excessive fibroproliferation responses, which are characterized by proliferation of fibroblasts cells with deposition of extracellular matrix, resulted in maladaptive lung repair and ultimately in lung fibrosis.^9^, ^10^, ^11^ Therefore, a novel therapeutic approach with the goal of stimulating migration, proliferation and differentiation of AT2 cells and mitigating aberrant fibroproliferative responses may be a promising way to attenuate lung injury and fibrosis.

Protectin DX (10S,17S‐dihydroxydocosa‐4Z,7Z,11E,13Z,15E,19Zhexaenoic acid) (PDX), an endogenous lipid mediator, derived from natural ω‐3‐fatty acid docosahexaenoic acid (DHA), displays anti‐inflammatory and potent inflammation pro‐resolving actions.^12^ Our previous studies have revealed that PDX maintains the integrity of lung epithelium, increases alveolar fluid clearance and ameliorates interstitial fibrosis.^13^, ^14^ Recently, PDX was also reported to inhibit PMN infiltration into the alveolar space,^15^ promote M2 polarization,^16^ accelerate resolution of inflammation,^16^ attenuate insulin resistance^17^ and decrease ROS production from neutrophils.^18^ However, the effects of PDX on epithelial injury repair and fibroproliferation remain ambiguous.

In this study, we aimed to investigate whether PDX has anti‐inflammatory effect through modulating alveolar epithelial cell injury repair and apoptosis. Also, we aimed to determine whether PDX affects fibroproliferation and fibroblast activation. Our results suggest that PDX promotes epithelial cell injury repair and inhibits epithelial cell apoptosis in vivo and in vitro. Also, PDX inhibits TGF‐β_1_ induced fibroproliferation, fibroblast collagen production and myofibroblast transformation. Finally, we provide evidence for a potential of an ALX receptor and PI3K signalling pathway in epithelial wound healing, fibroblast proliferation and activation.

## 2 MATERIALS AND METHODS

### 2.1 Materials

All the materials used in the manuscript were listed in Table S1.

### 2.2 Ethics

All procedures in this study were performed in accordance with the Guide for the Care and Use of Laboratory Animals. The study was approved by the Committee of Animals studies of Wenzhou Medical University.

### 2.3 Stimuli and inhibitors

**FIGURE 1:**
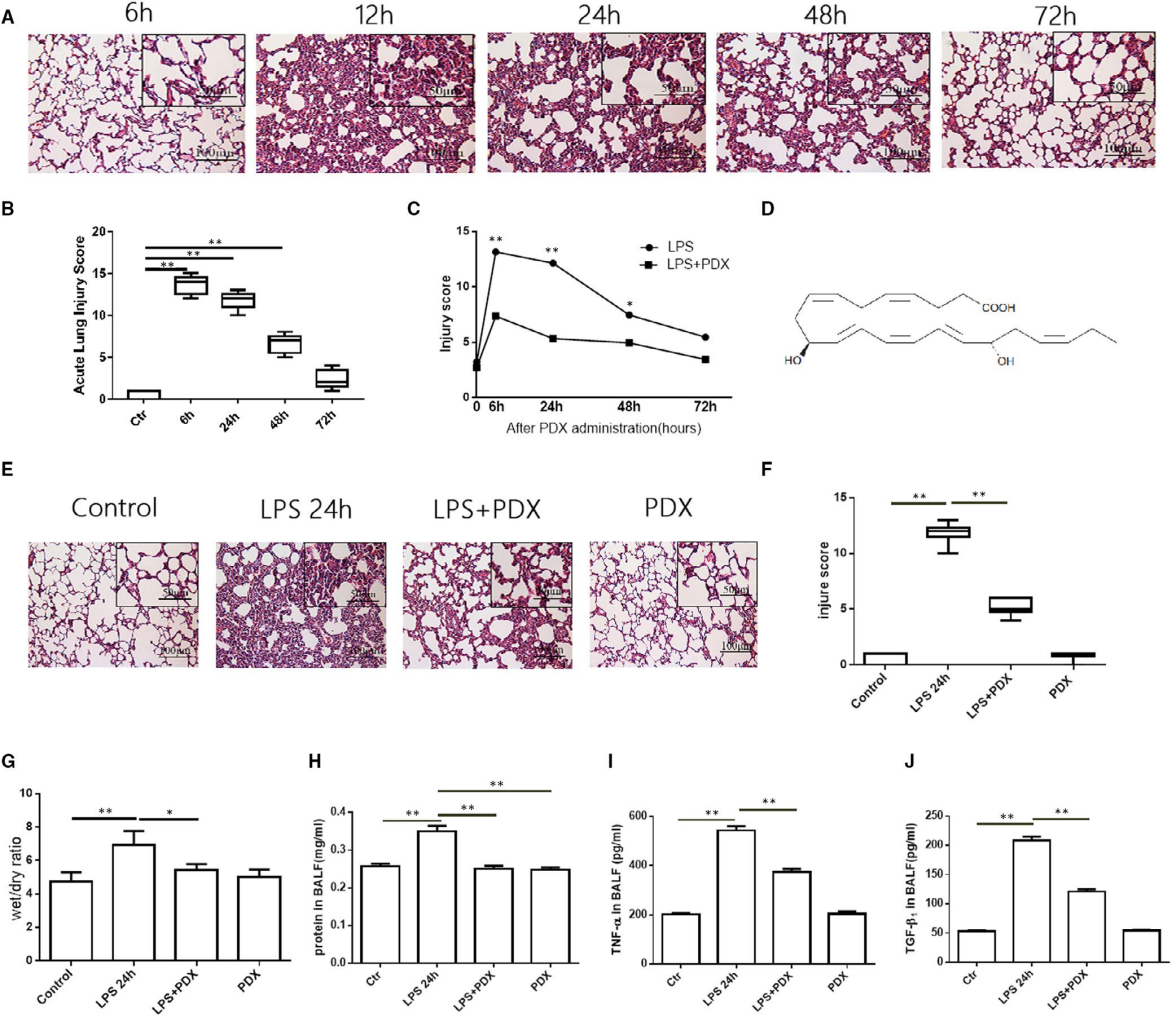
Protectin DX promoted the resolution of inflammation in the intratracheal LPS murine model of ALI/ARDS. A, LPS (10 mg/kg) was endotracheal given for 6 h, 24 h, 48 h and 72 h in mice. Control mice were treated with equal volume of saline. B, Injury score of lung section. C, N.S 30 μL or LPS 10 mg/kg in 30 μL was endotracheal given for 6 h, 24 h, 48 h and 72 h, with or without PDX 200 ng in 0.1 mL N.S or 0.1 mL N.S intraperitoneal injection per mouse. Data were presented with means ± SD. **P < .01; *P < .05; (LPS versus LPS + PDX). D, Chemical structure of PDX. E, Mice received endotracheal LPS (10 mg/kg), with or without PDX 200 ng for 24 h. Control mice were treated with equal volume of saline. F, Injury score of lung section. G, Wet/dry ratio of lung. H, Protein concentration in BALF. I, Concentration of TNF‑α. J, Concentration of TGF‐β_1_. Data were presented with means ± SD. *P < .05, **P < .01. n = 6; BALF, bronchoalveolar lavage fluid; PDX, Protectin DX; TNF‑α, tumour necrosis factor‑α; TGF‐β_1_, transforming growth factor‐β_1_

Protectin DX was purchased from Cayman Chemical. The chemical structure of PDX was shown in Figure 1D. PDX was dissolved in ethanol as supplied by the manufacturer and was stored at −80°C. Before use, ethanol was evaporated under a gentle stream of nitrogen; then, PDX was dissolved immediately in sterile saline or culture medium to the desired concentrations. Inhibitors were used at the following concentrations according to manufacturer's instructions: LY294002 (a PI3‐kinase inhibitor) at 10 µmol/L, and BOC‐2 (N‐t‐Boc‐Phe‐Leu‐Phe‐Leu‐Phe, ALXR antagonist) at 10 µmol/L. Cells were incubated with inhibitors for 1 h prior to every treatment. Appropriate vehicle controls were used for all experiments with inhibitors.

### 2.4 Animal preparation

Six‐ to 8‐week‐old C57BL/6 mice weighing 23‐25 g were obtained from Slac Laboratory Animal Co. Ltd. Before the experiments, all mice were housed in a specific pathogen‐free room with controlled temperature (22‐24°C) and humidity (50‐60%) under a 12 h day–night cycle. The mice were given standard laboratory chow and water ad libitum.

### 2.5 Animal model of ARDS/ALI

Mice were randomly divided into four groups (n = 6): control, LPS, LPS plus PDX (LPS + PDX) and PDX. The lung injury/ARDS model of mice was created by endotracheal injection of LPS (Escherichia coli 055: B5, Sigma‐Aldrich) (10 mg/kg) for 6 hours, 24 hours, 48 hours and 72 hours, with high‐pressure air pipe spray needle under direct vision. Equivalently sterile saline was injected to control and PDX group as solvent control. PDX was intraperitoneally administered, 200 ng/per mouse. Then, the mice were sacrificed, and the lungs and bronchoalveolar lavage fluid (BALF) were rapidly collected for further use. No death was observed in LPS‐treated mice.

### 2.6 Haematoxylin and eosin (H&E)

The lung lobes were fixed in 4% paraformaldehyde for 24 hours and completely embedded in paraffin. Lung tissues were cut into sections and stained with haematoxylin and eosin (H&E) for light microscopy analysis. Lung injury pathological scores were evaluated according to the study of C S et.al.^19^, ^20^

### 2.7 The protein concentration and cytokine (TNF‐α, TGF‐β_1_) levels in bronchoalveolar lavage fluid

Bronchoalveolar lavage fluid (BALF) collection is as described in Appendix S1. The protein concentration in BALF was measured by the BCA method. TNF‐α and TGF‐β_1_ were measured using enzyme‐linked immunosorbent assay kits (eBioscience Co) according to the manufacturer's instructions.

### 2.8 Immunofluorescence

Lung tissues were fixed and stained as described in Data S1.

### 2.9 Quantitative real‐time PCR and reverse transcriptase PCR

Total RNA samples were extracted from primary AT II cells and fibroblasts using Trizol reagent. cDNA was synthesized from 1 µg total RNA using the RT reagent kit. qPCR was performed using SYBR Green super‐mix PCR kit. Details of PCR primers are outlined in Table S2. Relative mRNA amounts were calculated using the 2^−∆∆CT^ method.^21^

### 2.10 Flow cytometry (FCM)

AT II cells were left in serum‐free media for 24 hours before treated by LPS (1 μg/mL) with or without PDX 100 nmol/L for 24 hours. After treatment of LPS and PDX, AT II cells were washed with cold PBS twice and then digested by 0.25% trypsin for 5 minutes until the cells were to drift. Followed by serum neutralization and washing with PBS containing 1% FBS and 0.05% EDTA twice and then stained with Annexin V‐FITC and PI according to manufacturer's recommendations. The percentage of cells in Q1‐UR and Q1‐LR were considered the apoptosis percentage in each experiment.

### 2.11 In vitro alveolar epithelial wound repair assay

The alveolar epithelial wound repair was performed as described before.^22^

### 2.12 Cell proliferation assay and cell viability assay

The cell proliferation and cell death assays were determined as described before. ^23^ The details are shown in Data S1.

### 2.13 Cell culture

Rat AT II cells and fibroblasts were extracted and cultured as according to published procedures.^24^, ^25^ Briefly, rat lung being instilled was finely minced, and following digested by dispase (5 U/mL) and DNase I (2 U/µL) at 37°C for 45 minutes in the incubator. Dissociated cells were then pushed through a 100 micron cell strainer, followed by a 40 micron cell strainer to remove connective tissue. Then, cells were lysed by RBC lysis buffer at room temperature for 30 minutes. The suspension was centrifuged, and the cells were resuspended in 50:50 DMEM/F‐12 media. The cells were plated on dual antibody coated plates prepared 24 hours in advance with anti‐rat CD16/CD32 and anti‐rat CD45.1. After incubation for 2 hours at 37°C to facilitate negative selection for type II alveolar cells, the media containing the suspended type II alveolar cells was removed and plated in the plates. The average purity of primary rat alveolar type II cells was 90%‐95% ATII‐like cells. Cells were tested for primary rat alveolar type II (AT II) cell phenotype by alkaline phosphatase staining, lysotracker lamellar body staining and by electron microscopy (sem) (data not shown).

The pulmonary fibroblasts were isolated from lung of the rat. Lung tissue was cut into <1 mm^3^ pieces and dissociated in Hanks buffered saline solution (HBSS) containing 0.25% trypsin at 37°C for 3 minutes. Trypsin was inhibited by DMEM with 10% FBS and dissociated tissue centrifuged at 500 g for 5 minutes at 4°C. The dissociated tissue pieces were then digested by type IV collagenase in a 37°C shaker for 2 hours, then neutralized with 10% DMEM and centrifuged again as before. Then, they were placed into a glass culture plate with DMEM containing 15% FBS and left to allow fibroblast outgrowth. After fibroblasts had grown out from the tissues, usually 3‐5 days, the remaining tissue was removed by aspiration, and the cells were allowed to reach confluence. Confluent fibroblasts were then passaged with a split ratio of 1:2 by trypsin treatment and used for the experiments at passages 4‐6.

### 2.14 Western blot analysis

Western blot analyses were determined as described previously.^25^ After equal amounts of protein were electrophoresed on 10% SDS‐PAGE gels and then transferred to PVDF membranes (Millipore, Billerica MA01821). Membranes were incubated with the indicated primary antibody overnight at 4°C. Then, membranes were incubated with appropriate secondary antibodies for 1 h. Western blot analysis was determined by the Image Quant LAS 4000 mini (GE).

### 2.15 Statistical analysis

Data are expressed as means ± standard deviations (SD) or means ± standard errors of the means (SEM). All data were normally distributed and analysed using one‐way ANOVA, followed by a Tukey test for multiple comparisons using Prism 6.0 software (GraphPad Software). *P* ≤ .05 was assumed to represent a statistically significant difference.

## 3 RESULTS

### 3.1 Protectin DX promoted the resolution of inflammation in the intratracheal LPS murine model of ALI/ARDS

First, we established LPS‐induced ALI/ARDS in mouse. The mice received LPS for 6 hours, 24 hours, 48 hours and 72 hours (Figure 1A). Compared with control group, LPS 6 hours, 24 hours and 48 hours groups showed markedly damaged pulmonary architecture with increasing in lung injury scores (Figure 1A,B). As time went by, the damaged pulmonary architecture decreased indicating the resolution of inflammation. Then, we evaluated the effect of PDX on the intratracheal LPS murine model of ALI/ARDS. LPS was endotracheal given for 6 hours, 24 hours, 48 hours and 72 hours, with or without PDX. LPS‐induced histologic signs markedly alleviated at 6 hours, 24 hours and 48 hours treated by PDX consistent with a decrease in lung injury scores and biggest decrease occurred both at 6 hours and at 24 hours (Figure 1C,E,F). In accordance with this, PDX also alleviated pulmonary permeability by relieving wet/dry ratio of lung tissue at 24 hours (Figure 1G). PDX dramatically decreased the protein concentration in BALF induced by LPS at 24 hours (Figure 1H). Cellular inflammation and pulmonary permeability were also elevated in LPS group, along with release of TNF‐α and TGF‐β_1_ (Figure 1I,J). However, treatment with PDX notably diminished the release of TNF‐α and TGF‐β_1_ at 24 hours (Figure 1I,J). No significant difference was found between the control and PDX groups.

### 3.2 PDX promoted type II alveolar epithelial cells proliferation while reduced type II alveolar epithelial cells apoptosis during acute lung injury induced by LPS in vivo

**FIGURE 2:**
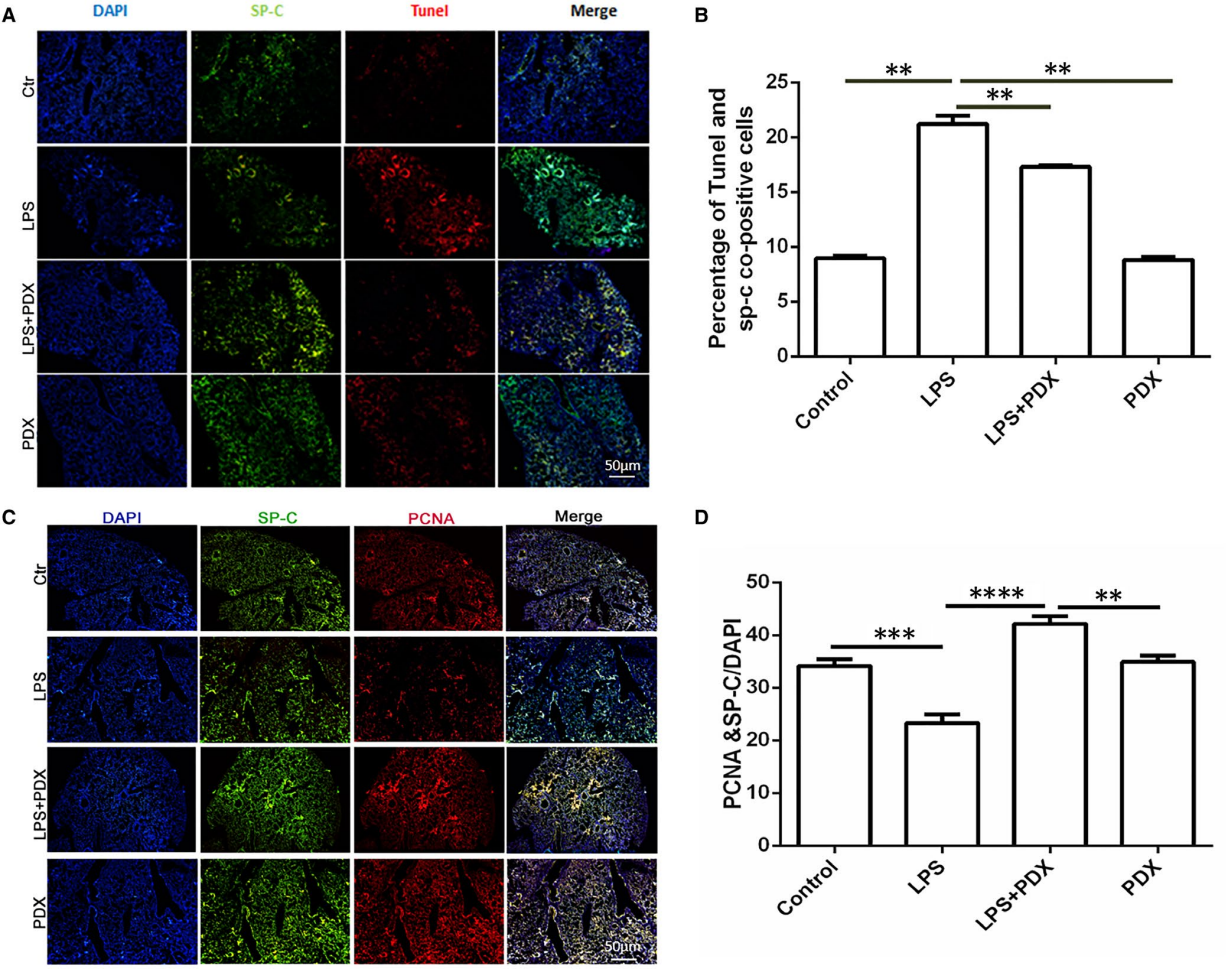
PDX promoted type II alveolar epithelial cells proliferation while reduced type II alveolar epithelial cells apoptosis during acute lung injury induced by LPS in vivo N.S 30 μL or LPS 10 mg/kg in 30 μL was endotracheal given for 24 h, with or without PDX 200 ng in 0.1 mL N.S or 0.1 mL N.S intraperitoneal injection per mouse. A and C, Immunofluorescence of AT II cells (×100, scar bar = 100 μm). B, The quantization of apoptosis of AT II cells. D, The quantization of proliferation of AT II cells. Data were presented with means ± SD; n = 6; **P < .01

In vivo, to determine whether PDX affects the proliferation and apoptosis of type II alveolar epithelial cells in mice, lung specimens SP‐C (a type II cell marker) and PCNA or SP‐C and TUNEL double‐staining immunofluorescence were observed, respectively (Figure 2). PDX significantly promoted the proliferation of type II alveolar epithelial cells (SP‐C/PCNA double positive) in LPS‐induced lung injury (Figure 2). While comparing with the control group, LPS induced the apoptosis of AT II cells, and PDX inhibited the effect of LPS on the apoptosis of AT II cells in vivo (Figure 2).

### 3.3 PDX stimulated primary rat AT II cells wound repair and proliferation partly via the ALX/PI3K signalling pathway

**FIGURE 3:**
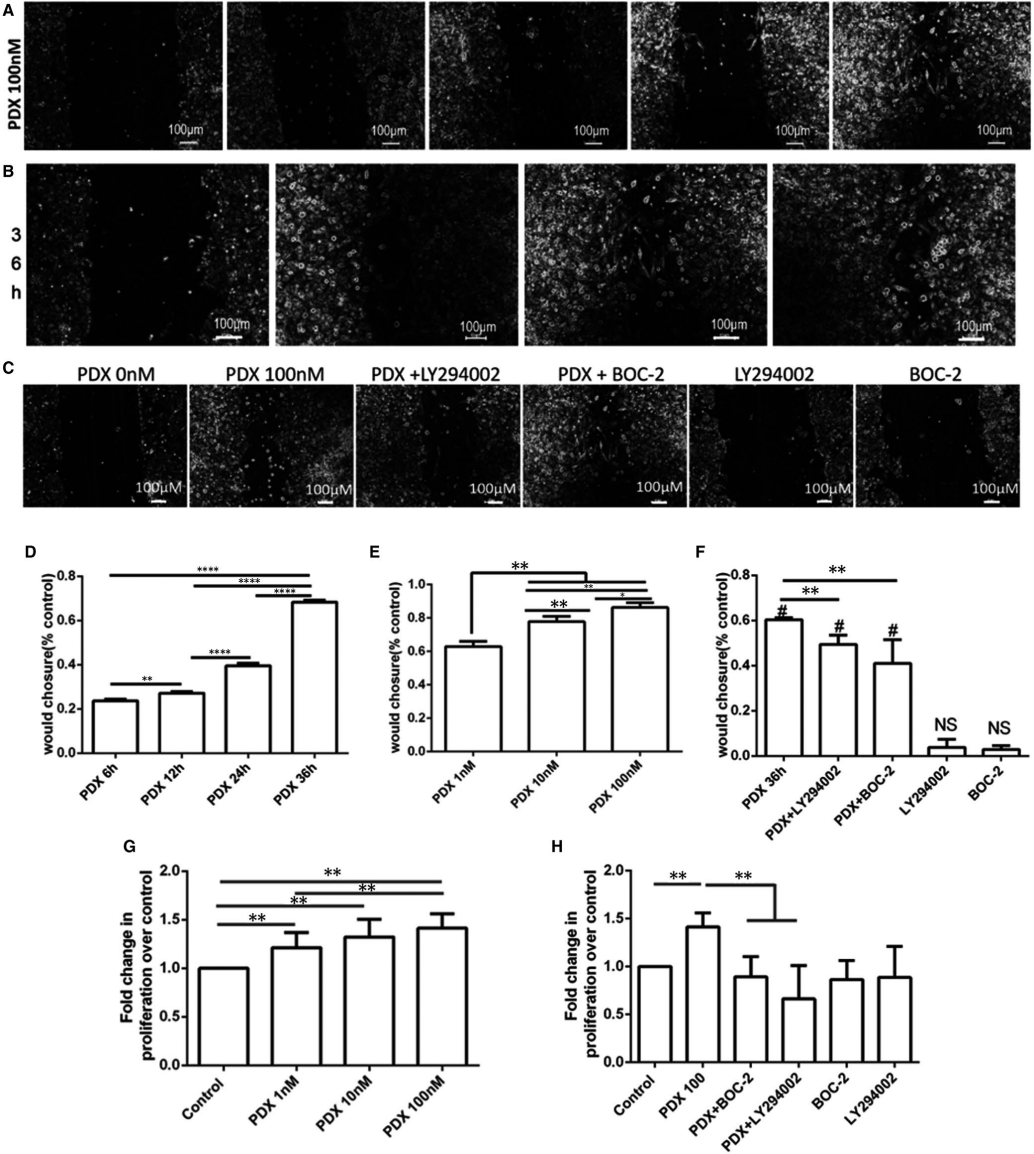
PDX stimulated primary rat AT II cells wound repair and proliferation partly via the ALX/PI3K signalling pathway A and D, Time‐dependent effect of PDX on AT II cells would repair. B and E, Dose‐dependent effect of PDX on AT II cells wound repair. G, PDX from 1 nmol/L to 100 nmol/L was incubated with primary AT II cells for 24 h. PDX stimulated the proliferation of primary AT II cells in a dose‐dependent manner. C, F and H, LY294002 and BOC‐2 were re‐incubated with primary AT II cells at 10 μmol/L for 1 h before the addition of PDX 100 nmol/L. The effect of BOC‐2 and LY294002 on PDX stimulated AT II wound repair and proliferation. The wound closure pictures were taken by the microscope. Data were presented as mean ± SEM of three independent experiments. *P < .05; **P < .01

We further investigate whether PDX affects the scratch wound closure and the proliferation of rat primary AT II cells. We found that PDX stimulated primary rat AT II cells wound repair in a time‐ and dose‐dependent manner (Figure 3A,B,D,E). Scratch wound repair could occur because of either proliferation and/or spreading of cells. Therefore, we confirmed if PDX can affect ATII cells proliferation. We found PDX dose dependently promoted proliferation of primary rat AT II cells (Figure 3G).

To investigate whether PDX promotes wound closure and proliferation of primary rat type II alveolar epithelial cells via ALX/PI3K signalling pathway, primary rat AT II cells were incubated with BOC‐2(10 μmol/L) and LY294002(10 μmol/L) 1 hour prior to PDX treatment (Figure 3C,F,H). As expected, the beneficial effects of PDX on AT II cells wound repair and proliferation were blocked by treatment with BOC‐2 and LY294002 (Figure 3C,F,H).

### 3.4 PDX inhibited primary rat type II alveolar epithelial cells death induced by LPS

**FIGURE 4:**
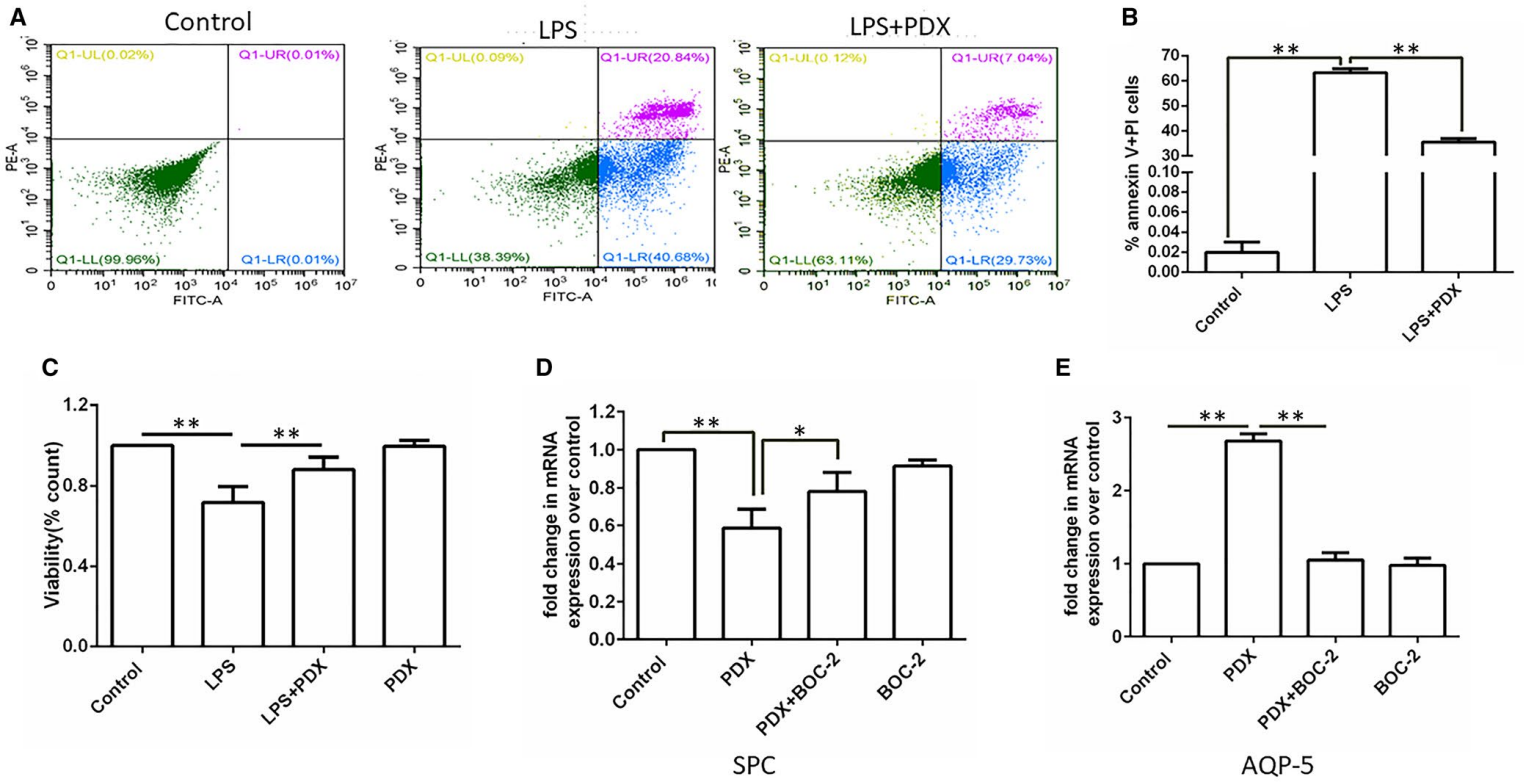
Effects of PDX on LPS stimulated (AT II) cells death and transdifferentiation. Primary ATII cells treated with 1 μg/mL LPS or/and PDX at 100 nmol/L for 24 h. A and B, Flow cytometry plots of different groups. After treatment, ATII cells were stained with Annexin V‐FITC and PI according to manufacturer's recommendations. The percentage of cells in Q1‐UR and Q1‐LR were considered the apoptosis percentage in each experiment. C, The viability of AT II cells. D, The mRNA expression of SP‐C; E, The mRNA expression of AQP‐5. Data were presented with mean ± SEM of three independent experiments. *P < .05；**P < .01

To confirm the protective effect of PDX on the apoptosis of AT II cells, we further determined primary rat type II alveolar epithelial cells death induced by LPS. As data presented in Figure 4A,B, the apoptosis percentage of primary rat AT II cells in the LPS group was 61.52%, reduced to 36.77% in LPS + PDX group. Furthermore, detection of the viability of AT II cells verified this result (Figure 4C).

### 3.5 PDX promoted aquaporin 5 gene expression while reducing SP‐C gene expression in primary rat type II alveolar epithelial cells

To investigate the effect of PDX on a type I epithelial cell marker of aquaporin 5 (AQP5) and a type II epithelial cell marker of Surfactant Protein C (SP‐C), PDX 100 nmol/L was incubated with primary rat type II alveolar epithelial cells for 24 hours. PDX up‐regulated AQP5 gene expression, and this effect was abrogated by BOC‐2 (Figure 4E). In comparison, PDX reduced SP‐C gene expression suggesting that PDX might promote transdifferentiation of type II epithelial cells into type I epithelial like cells (Figure 4D).

### 3.6 PDX inhibited proliferation of fibroblast in response to TGF‐β_1_ through the ALX/PI3K signalling pathway

**FIGURE 5:**
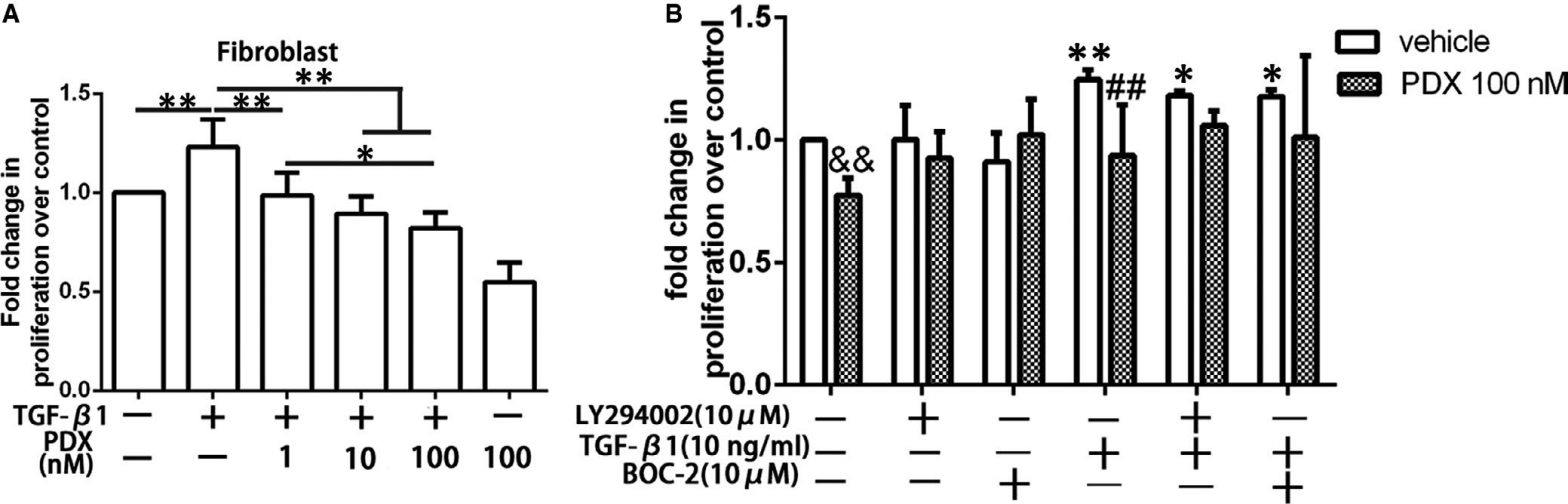
PDX inhibited proliferation of fibroblast in response to TGF‐β_1_ through the ALX/PI3K signalling pathway. A, Primary fibroblasts were incubated with 10 ng/mL TGF‐β_1_ and (or) PDX from 1 nmol/L to 100 nmol/L for 24 h. B, Cultured and serum‐deprived cells were treated with 10 ng/mL TGF‐β_1_ for 24 h with or without pre‐incubation with LY294002 (10 μmol/L) or BOC‐2 (10 μmol/L) for 1 h. Data were presented with mean ± SEM of three independent experiments. &&*P* < .01, **P < .01 compared with no treatment group；*P < .05, ^##^ *P* < .01, compared with TGF‐β_1_ only group

Fibroblast proliferation studies in vitro showed that PDX suppressed TGF‐β_1_ induced primary fibroblasts proliferation in dose‐dependent manner (Figure 5A). Furthermore, to determine the ALX/PI3K‐dependent actions of PDX on fibroblast proliferation, primary fibroblasts were incubated with BOC‐2 (10 μmol/L) and LY294002 (10 μmol/L) 1 hour prior to the treatment of PDX. The results verified that the effect of PDX on fibroblasts was partly via ALX/PI3K signalling pathway (Figure 5B).

### 3.7 PDX down‐regulated collagen production of fibroblast and differentiation to myofibroblast through ALX/PI3K signalling pathway

**FIGURE 6:**
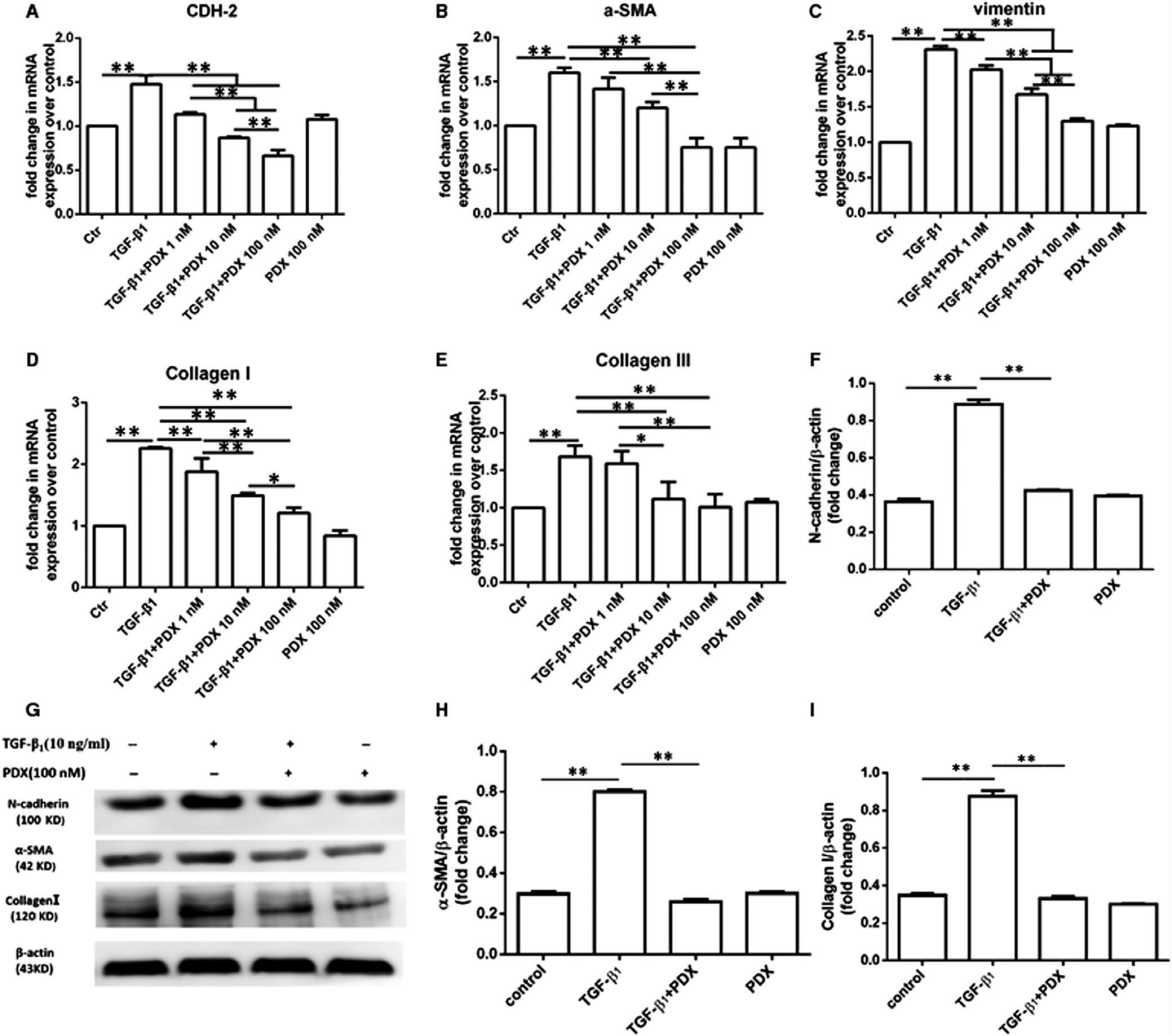
PDX inhibited fibroblast activation to myofibroblast and reduced the deposition of collagen. Primary fibroblasts were incubated with 10 ng/mL TGF‐β_1_ and (or) PDX from 1 nmol/L to 100 nmol/L for 24 h. A, The mRNA expression of N‐cadherin (CDH‐2); B, the mRNA expression of α‐SMA; C, the mRNA expression of vimentin; D, the mRNA expression of type I collagen; E, the mRNA expression of type III collagen. F‐I, The effects of PDX on the TGF‐β_1_‐treated N‐cadherin, α‐SMA, type I collagen of ATII cells were confirmed by western blot. Data were presented with mean ± SEM of three independent experiments. *P < .05, **P < .01

**FIGURE 7:**
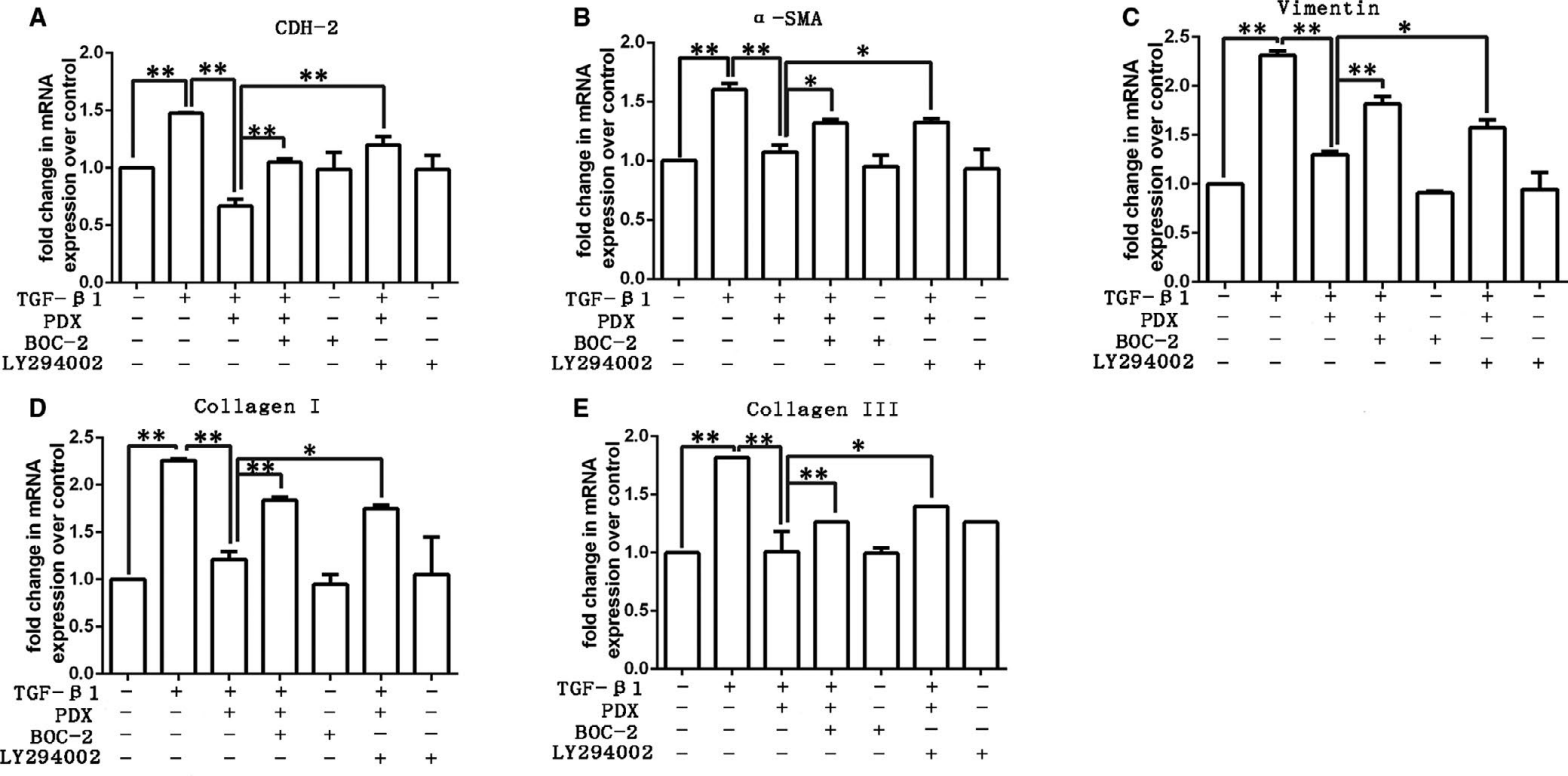
The mechanism of PDX inhibited fibroblast activation to myofibroblasts and reduced the deposition of collagen. LY294002 (10 μmol/L), BOC‐2 (10 μmol/L) and PDX 100 nmol/L were added 1 h prior to TGF‐β_1_ (10 ng/mL). A, The mRNA expression of N‐cadherin (CDH‐2); B, the mRNA expression of α‐SMA; C, the mRNA expression of vimentin; D, the mRNA expression of type I collagen; E, the mRNA expression of type III collagen. Data were presented with mean ± SEM of three independent experiments. * *P* < .05, ** *P* < .01

We determined whether PDX affects gene expression of type I collagen, type III collagen, N‐cadherin, α‐SMA and vimentin in primary rat fibroblasts. As shown in Figure 6, TGF‐β_1_ increased the mRNA expression of type I collagen, type III collagen, N‐cadherin, α‐SMA and vimentin. PDX reduced the mRNA expression of type I collagen, type III collagen, N‐cadherin, α‐SMA and vimentin in dose‐dependent manner, respectively (Figure 6A‐E). Western blot was used to confirm the effects of PDX on the protein expression of N‐cadherin, α‐SMA and type I collagen induced by TGF‐β1 in primary fibroblasts (Figure 6F‐I). Moreover, pre‐treatment with LY294002 and BOC‐2 abrogated the effects of PDX on the mRNA expression of type I collagen, type III collagen, N‐cadherin, α‐SMA and vimentin (Figure 7).

## 4 DISCUSSION

The present study showed that PDX promoted physical wound healing by inducing primary rat type II alveolar epithelial cells proliferation, while inhibiting LPS treated primary type II alveolar epithelial cells apoptosis in vitro. In vivo, we also demonstrated PDX increased type II alveolar epithelial cells proliferation and inhibited type II alveolar epithelial cells apoptosis, which attenuated lung injury induced by LPS. Intriguingly, PDX also inhibited TGF‐β_1_ induced fibroproliferation, fibroblast collagen production and myofibroblast transformation suggesting beneficial effect of PDX on abnormal fibroproliferative responses. In addition, BOC‐2 and LY294002 abrogated the effects of PDX on the proliferation, wound closure of AT II cells indicating that PDX promotes AT II cells injury repair to reduce lung injury via the ALX/PI3K signalling pathway. Also, PDX reduced TGF‐β_1_ induced proliferation and differentiation to myofibroblasts in primary lung fibroblast partly through the ALX/PI3K signalling pathway.

Clinically relevant E. coli lipopolysaccharide (bacterial cell wall products, LPS) challenge is a widely model mimicking acute respiratory distress syndrome/acute lung injury (ARDS/ALI).^26^ The studies reported that E. coli LPS was able to directly (eg pneumonia) and indirectly (eg sepsis) destroy alveolar epithelial cells thereby facilitating the disruption of the pulmonary epithelial barrier.^27^, ^28^ Therefore, in our study, intratracheal E. coli LPS was employed as a model of ARDS/ALI. In addition, we assessed if E. coli LPS would induce epithelial cells injury.

Epithelial injury in the lung is a hallmark of ALI/ARDS and fibrotic lung diseases. it has been established that epithelial repair and regeneration are crucial for ALI/ARDS resolution.^29^ In our intratracheal LPS murine model of ALI/ARDS, increased epithelial injury was evident. PDX stimulated type II alveolar epithelial cells proliferation and restored epithelial barriers. In vitro, we demonstrated that PDX promoted physical wound healing in a dose‐dependent manner. Scratch injury closure can happen due to proliferation and migration of cells. We also found that PDX promoted the primary AT II cells proliferation in a dose‐dependent manner as well. PI3K/AKT is a classical signal pathway that controls cell proliferation and differentiation.^30^ Furthermore, we also found that the mitogenic response of AT II cells to PDX is via the ALX/PI3K signalling pathway.

The restoration of the injury epithelial barrier is thought to involve the transdifferentiation of type II epithelial cells into type I epithelial like cells. AQP5 is an aquaporin and a marker of AT I cell, which was down‐regulated during lung injury.^31^ Our results demonstrated that PDX decreased the gene expression of SP‐C (AT II cell marker) and up‐regulated the gene expression of AQP5 through ALX receptor activation suggesting PDX might promote the transdifferentiation from type II epithelial cells into type I epithelial like cells and fluid transport.

Epithelial cell loss due to apoptosis represents a potentially important mechanism of ALI/ARDS.^32^ Alveolar epithelial cells apoptosis is believed to occur early in ALI/ARDS.^33^ The expression of TUNEL, a apoptotic marker, was up‐regulated in lung tissue from ALI/ARDS patients.^34^ The study reported that down‐regulation of apoptosis using a broad‐spectrum caspase inhibitor alleviated lung injury in mice induced by LPS.^35^ Our study also demonstrated that PDX reduced AT II cells apoptosis, which attenuated LPS‐induced lung injury in vivo, as well as in vitro. Therefore, early down‐regulation of apoptosis may be an effective strategy for the treatment of ALI/ARDS.

In our previous study, we demonstrated that PDX reduced lung fibrosis by inhibiting epithelial‐mesenchymal transition (EMT) in vivo, as well as in vitro.^13^ PDX up‐regulated alveolar fluid clearance and facilitates the resolution of inflammation in part through activation of ALX receptor.^14^ Herein, we found PDX decreased TGF‐β_1_ induced primary rat lung fibroblast proliferation which was mediated via ALX/PI3K signalling pathway. It is proved that TGF‐β_1_ is necessary to promote differentiation of fibroblasts to myofibroblasts,^36^, ^37^ which up‐regulated the levels of mesenchymal phenotype ( such as α‐SMA, N‐cadherin) and extracellular matrix proteins.^38^, ^39^ In our study, we also showed that TGF‐β_1_ expression in the BALF of LPS‐treated mice was notably augmented. Therefore, in our vitro study, fibroblast activation was induced with TGF‐β_1_ treatment. Our data showed PDX significantly suppressed TGF‐β_1_‐induced mRNA expression of type I collagen, type Ⅲ collagen, α‐SMA, N‐cadherin and vimentin in primary rat lung fibroblast. Western blot results also confirmed the effects of PDX on protein expression of N‐cadherin, α‐SMA and type I collagen in primary rat lung fibroblast. We further explored the mechanism of the effect of PDX on fibroblasts and found that it was partly through the ALX/PI3K pathway.

In conclusion, this study confirmed that PDX promoted AT II cells wound closure by inducing primary rat type II alveolar epithelial cells proliferation and inhibited LPS treated primary type II alveolar epithelial cells apoptosis in vivo and in vitro, which attenuated lung injury induced by LPS. PDX up‐regulated the mRNA expression of AQP5 and reduced the mRNA expression of SP‐C potentially promoting transdifferentiation, which plays a key role in restoration after alveolar injury. Furthermore, we also demonstrated PDX inhibited fibroblast proliferation, collagen production and expression of mesenchymal phenotype in response to TGF‐β_1_. This data presented identify a new mechanism by PDX which target the airway epithelial cell and fibroproliferation are potential for treatment of ALI/ARDS.

## CONFLICT OF INTEREST

The authors declare no conflict of interest.

## AUTHOR CONTRIBUTION

**Jing‐Xiang Yang:** Investigation (equal); Project administration (equal); Resources (equal); Validation (equal); Visualization (equal). **Ming Li:** Formal analysis (equal); Investigation (equal); Methodology (equal); Project administration (equal); Validation (equal); Visualization (equal). **Xin Hu:** Data curation (equal); Formal analysis (equal); Validation (equal); Visualization (equal). **Jia‐Chao Lu:** Formal analysis (equal); Methodology (equal); Project administration (equal); Validation (equal). **Shi‐Yue Lu:** Investigation (equal); Project administration (equal); Validation (equal). **qian wang:** Data curation (equal); Formal analysis (equal); Project administration (equal); Validation (equal). **Fang Gao Smith:** Conceptualization (equal); Data curation (equal); Supervision (equal); Validation (equal); Writing‐review & editing (equal). **Sheng‐Wei Jin:** Conceptualization (equal); Data curation (equal); Formal analysis (equal); Validation (equal); Visualization (equal); Writing‐original draft (equal); Writing‐review & editing (equal). **Sheng‐Xing Zheng:** Conceptualization (equal); Data curation (equal); Formal analysis (equal); Methodology (equal); Resources (equal); Supervision (equal); Validation (equal); Writing‐original draft (equal); Writing‐review & editing (equal).

## Supporting information

App S1

## Data Availability

Data that support the findings of this study are provided from the corresponding authors upon request.
