# Correction to “Protectin DX Promotes Epithelial Injury Repair and Inhibits Fibroproliferation Partly via ALX/PI3K Signalling Pathway”

**DOI:** 10.1111/jcmm.71087

**Published:** 2026-03-10

J. X. Yang, M. Li, X. Hu, et al., “Protectin DX Promotes Epithelial Injury Repair and Inhibits Fibroproliferation Partly via ALX/PI3K Signalling Pathway,” *Journal of Cellular and Molecular Medicine* 24, no. 23 (2020): 14001–14012, https://doi.org/10.1111/jcmm.16011

In Yang JX et al., Figure 3 was incorrect due to an error in the preparation of this figure for publication. In Figure 3B, Figure 3C of the published article, panels from the PDX 36h group were incorrectly placed to represent the PDX 10nM group and the PDX+BOC‐2 group separately due to mistakes during figure assembly. The corrected Figure 3 appears below. We apologize for any confusion this may have caused.

**FIGURE 3:**
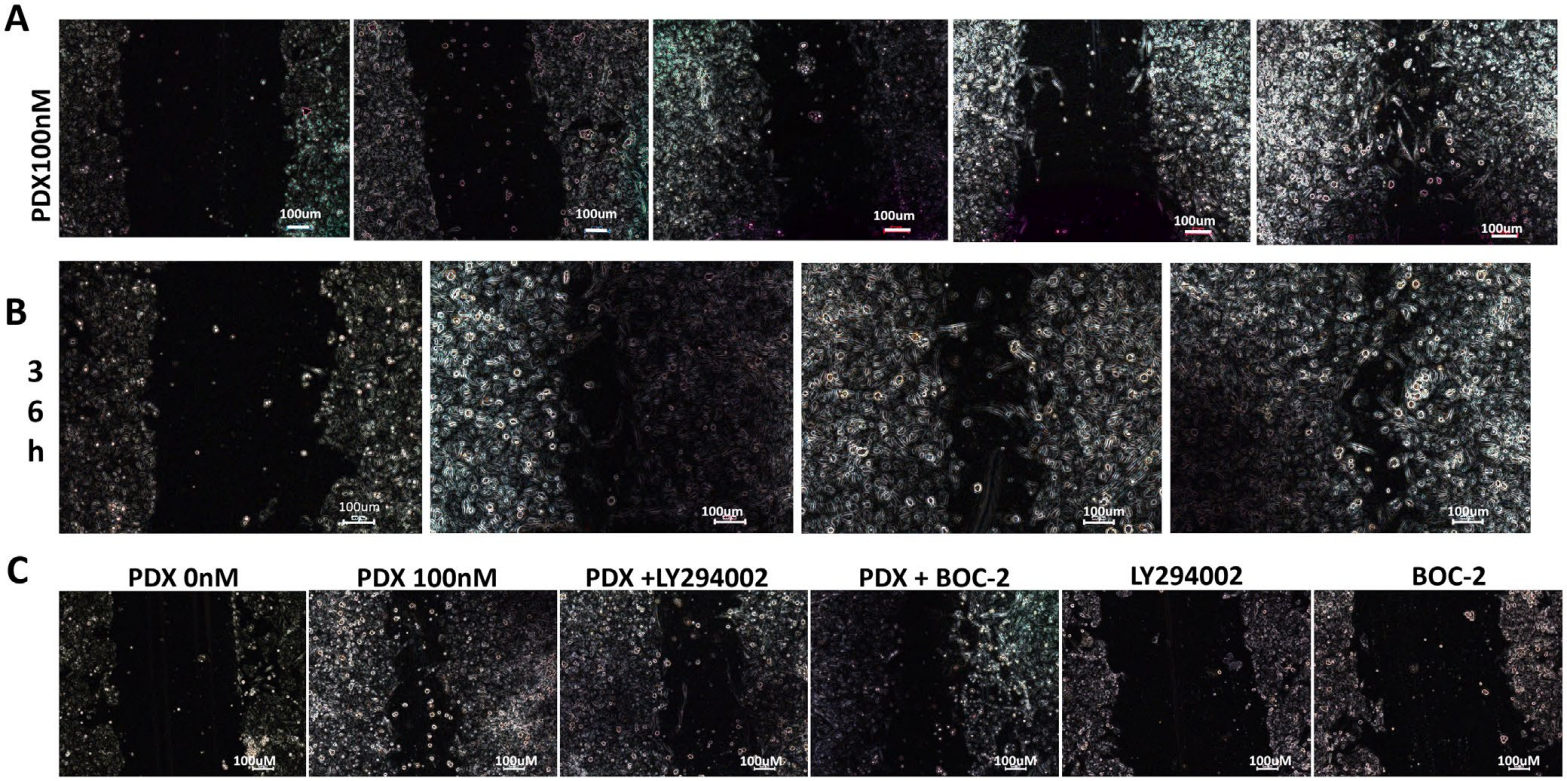
PDX stimulated primary rat AT II cells wound repair and proliferation partly via the ALX/PI3K signaling pathway.

