# Supplementary material for: Protectin DX promotes epithelial injury repair and inhibits fibroproliferation partly via ALX/PI3K signalling pathway
Source: J Cell Mol Med. 2020 Oct 24;24(23):14001–12. doi: 10.1111/jcmm.16011 (PMC7754026; doi:10.1111/jcmm.16011)
Supplement: Supplementary file 1 — App S1 [file JCMM-24-14001-s001.docx]

**Supplementary materials and methods**

**Histological analysis of lung tissues**

For histological examinations, lung lobes of each animal were harvested, fixed in 4% paraformaldehyde for 24 h, embedded in paraffin wax, sectioned, and stained with H&E for light microscopy analysis. Lung injury scores were quantified by an investigator who was blinded to the treatment groups using an established histopathological scoring system which included alveolar congestion, alveolar hemorrhage, neutrophil infiltration, or aggregation in the airspace or vessel wall, and alveolar wall/hyaline membrane thickness and inflammatory cell infiltration. The grading scale for the light microscopy pathologic findings was as follows: 0 = no injury; 1 = slight injury (25%); 2 = moderate injury (50%); 3 = severe injury (75%); and 4 = very severe injury (almost 100%). The results were graded from 0 to 4 for each item, as described previously [1-2]. The four variables were summed to represent the lung injury score (total score: 0–16).

**Bronchoalveolar lavage fluid (BALF) collection**

At the end of the experimental period, animals were anesthetized. The trachea was exposed and cannulated by a catheter and BALF was collected by injections and aspiration of 2ml phosphate buffer saline (PBS) 3 times (total volume 6 ml). The BALF was centrifuged at 2500 rpm, 4 °C for 10 min. The supernatant of BALF was used for protein and cytokine assessment.

**Evaluation of Pulmonary edema**

The lower lobes of the right lung from each group were assessed for wet/dry weight ratios. The lungs were weighed, then placed in an oven for 72h at 60°C until weight was no longer changed. The dry lungs were weighted and the wet-to-dry (W/D) ratio was calculated.

**Immunofluorescence**

For lung section, after paraffin removal in xylene, the sections were rehydrated and placed in a pressure cooker with citrate buﬀer, pH 6.0, for 1 min after reaching boiling temperature to retrieve antigenic sites masked by formalin fixation. sections were incubated with antibody to SP-C, and PCNA in 1:100 dilution, TUNEL as the manufacturers’ instructions and with the pre-immune serum as a negative control stain overnight at 4 °C, and subsequently were incubated for 1 h at room temperature with the donkey anti-secondary antibodies: Alexa Fluor 488 or Alexa Fluor 594 and DAPI. Images were obtained using a 5000B Leica microscope equipped with a charge-coupled device camera interfaced with Q-Capture Pro software. Images were globally adjusted to optimize contrast and brightness, and composed using Image J. The quantitation of them were calculated by positive/DAPI.

**In vitro alveolar epithelial wound repair assay**

Epithelial repair was determined using an in vitro epithelial wound repair assay as described before [3]. Briefly, primary rat ATII cells were grown to confluent monolayers before wounding with a 1-ml pipette tip. Digital images of the same point on the wound were taken at time 0h, 6h, 12h, 24h and 36h. To control for the inconsistencies in wound size, only monolayers in which the original wound areas varied by 10% of the mean were analyzed. Repair is expressed as the percentage of the original wound area covered by cells relative to control media. To allow for variability between cell types and batches, data are expressed as the mean ± SEM percentage of control.

**Cell proliferation assay and cell viability assay**

Bromodeoxyuridine (Brd U) incorporation was assessed according to manufacturers’ instructions. Cell viability after 24h was assessed adding 20 μl of Cell Titer 96 aqueous one solution cell proliferation solution to cells for 1.5h at 37°C and 5% CO2. Data from proliferation bioassays comparing the Cell Titer 96 Aqueous Assay and hydrogen-3–thymidine incorporation show similar results [4].

**Cell cultures**

Type II alveolar cells were isolated according to published procedures [5]. Briefly, rat lung being instilled were finely minced, and following digested by 60 µL dispase (5 U/mL) and 7.5 µL DNase I (2 U/µL) per lung at 37 °C for 45 min in the incubator. Dissociated cells were then pushed through a 100 micron cell strainer, followed by a 40 micron cell strainer to remove connective tissue. After that, cells were lyase by RBC lysis buffer at room temperature for 30 min. The suspension was centrifuged and the cells were resuspended in 50:50 DMEM/F-12 media. The cells were plated on dual antibody coated plates prepared 24 h in advance with anti-rat CD16/CD32 and anti-rat CD45.1. After incubation for 2 hours at 37 °C to facilitate negative selection for type II alveolar cells, the media containing the suspended type II alveolar cells was removed and plated in the plates. The average purity of primary rat alveolar type II cells was 90-95% ATII-like cells. Cells were tested for primary rat alveolar type II (AT II) cell phenotype by alkaline phosphatase staining, lysotracker lamellar body staining and by electron microscopy (sem) (data not shown)

The pulmonary fibroblasts were isolated from lung of the rat [6]. Lung tissue was cut into <1 mm^3^ pieces and dissociated in Hanks buffered saline solution (HBSS) containing 0.25% trypsin at 37°C for 3 minutes. Trypsin was inhibited by DMEM with 10% FBS and dissociated tissue centrifuged at 500 g for 5 minutes at 4°C. The dissociated tissue pieces were then digested by type IV collagenase in a 37 ℃ shaker for 2h，then neutralized with 10% DMEM and centrifuged again as before. Then placed into a glass culture plate with DMEM containing 15% FBS and left to allow fibroblast outgrowth. After fibroblasts had grown out from the tissues, usually 3-5 days, the remaining tissue was removed by aspiration, and the cells were allowed to reach confluence. Confluent fibroblasts were then passaged with a split ratio of 1:2 by trypsin treatment and used for the experiments at passages 4–6. Fibroblasts were also second purified by the time difference between the sidewall time through medium change after being passaged 45min.

For all experiments, cells were plated in plates and grown to 80% confluence. Then, cells were serum deprived for 24 hours in DMEM medium containing 0.5% FBS prior to the addition of drugs.

**Stimuli and inhibitors**

AT II cells and fibroblasts were treated with PDX, LPS or TGF- β1 as described in every figure legend. Inhibitors were used at the following concentrations according to manufacturers’ instructions: LY294002, a PI3-kinase inhibitor at 10 μM; and the ALX receptor (ALXR) antagonist, BOC-2 (N-t-Boc-Phe-Leu-Phe-Leu-Phe; GenScript USA Inc, Piscataway, NJ, USA), at 10 μM. Inhibitors were added to cells 1h prior to every treatment.

**Table. S1 The materials used in this article**

| **Name** | **Abbreviation** | **Product NO.** | **Manufacturer** | **Country** |
| --- | --- | --- | --- | --- |
| **In Situ Cell Death Detection Kit, POD** | TUNEL | 11684817910 | Roche | Switzerland |
| **2-(4-Amidinophenyl)-6-indolecarbamidine dihydrochloride** | DAPI | ab104139 | Abcam | UK |
| **Anti-SP1, C-Terminal antibody** | SP-C | SAB4502837 | Sigma | America |
| **Anti-PCNA antibody** | PCNA | Ab29 | Abcam | UK |
| **Alexa Fluor 594** | NO | ab150108 | Abcam | UK |
| **Alexa Fluor 488** | NO | ab150077 | Abcam | UK |
| **Anti-fluorescence decay sealer** | NO | S2100 | Solarbio | China |
| **lipopolysaccharide** | (Escherichia coli serotype 055:B5) | L2880 | Sigma | America |
| **Protectin DX** | PDX | 10008128 | Cayman | America |
| **N-t-Boc-Phe-Leu**  **-Phe-Leu-Phe** | BOC-2 | 73572-58-4 | GenScript | America |
| **LY294002** | LY294002 | S1105 | Selleck | America |
| **Trypsin-EDTA (1X), Phenol Red** | Trypsin | 25200072 | Gibco | America |
| **Fetal calf serum** | FBS | 10099141 | Gibco | Australia |
| **Annexin V-FITC/PI apoptosis kits** | NO | 70-AP101-60 | MULTI SCIENES | China |
| **Anti-α-Smooth muscle actin Antibody** | α-SMA | 48938 | Cell Signal Technology | USA |
| **Anti- N-cadherin Antibody** | CDH2 | ab18203 | Abcam | UK |
| **Anti-Collagen I Antibody** | COLІ | ab34710 | Abcam | UK |
| **Anti-beta Actin Antibody** | β-actin | ab8227 | Abcam | UK |
| **BrdU Cell Proliferation Assay Kit** | Brd U | K306-1000 | Biovision | America |
| **Transforming**  **growth factor-beta-1 ELISA kit** | TGF-β_1_ | 384606 | eBioscience | USA |
| **Tumor necrosis factor alpha ELISA kit** | TNF-α | ab208348 | Abcam | UK |
| **RevertAid First Strand cDNA Synthesis Kit** | NO | K1622 | Thermo Fisher | America |
| **SYBR Green Realtime**  **PCR Master Mix-Plus** | SYBR | QPK-212 | TOYOBO | Japan |
| **TRIzol Regent** | Trizol | 15596018 | Invitrogen | America |

**Table. S2 Primers sequence of mRNA**

| **Gene name** | **Forward sequence(5’-3’)** | **Reverse sequence(5’-3’)** |
| --- | --- | --- |
| **Aquaporin-5** | GCCGTCAATGCGCTGAACAAC | CATGGAACAGCCGGTGAAGTAGATC |
| **SP-C** | TTGTCGTCGTGGTGATTGTAGG | GAAGGTAGCGATGGTGTCTGTG |
| **CDH-2** | GAGAGGAAGACCAGGACTATGA | TCTCGTCTAGCCGTCTGATT |
| **α-SMA** | CCGAGATCTCACCGACTACC | CCAGAGCGACATAGCACAG |
| **VIMENTIN (ACTA2)** | AGGGGAGGAGAGCAGGATT | GGAGTGGGTGTCAACCAGAG |
| **Collagen I** | TCTAAGACATCCCTGGTCAC | GTCCTTCCAGAAGAAACCTT |
| **Collagen III** | ACTGACCAAGGTAGTTGCATCCCA | CCAGGGTCACCATTTCTCC |
| **GAPDH** | ACAAGATGGTGAAGGTCGGTG | AGAAGGCAGCCCTGGTAACC |

1. C S, journal MMJTEr. Alveolar epithelial fluid transport in acute lung injury: new insights. 2002;20(5):1299-1313.
2. Shyamsundar M, McAuley DF, Ingram RJ, et al. Keratinocyte growth factor promotes epithelial survival and resolution in a human model of lung injury. Am J Respir Crit Care Med. Jun 15 2014;189(12):1520-1529.
3. Geiser T. <H2O2 inhibits alveolar epithelial wound repair in vitro. Am J Physiol Lung Cell Mol Physiol. 2004 2004; 287: :5.
4. A Z, EB T, RA W, et al. Comparison of 3H-thymidine incorporation and CellTiter 96 aqueous colorimetric assays in cell proliferation of bovine mononuclear cells. 1998;46(2):191-7.
5. Wang Q, Lian Q, Li R, et al. Lipoxin A(4) activates alveolar epithelial sodium channel, Na,K-ATPase, and increases alveolar fluid clearance. Am J Respir Cell Mol Biol. 2013;48(5):610-8.
6. Zheng SX, Wang Q, He Q, Song XL, Ye DY, Gao F, Jin SW, Lian QQ. Novel biphasic role of LipoxinA(4) on expression of cyclooxygenase-2 in lipopolysaccharide stimulated lung fibroblasts. Mediators Inflamm. 11(6):224-233, 2011.
